# Perioperative chemotherapy in the treatment of osteosarcoma: a 26-year single institution review

**DOI:** 10.1186/s13569-015-0032-0

**Published:** 2015-07-14

**Authors:** G M O’Kane, K A Cadoo, E M Walsh, R Emerson, P Dervan, C O’Keane, B Hurson, G O’Toole, S Dudeney, E Kavanagh, S Eustace, D N Carney

**Affiliations:** Department of Medical Oncology, Mater Misericordiae University Hospital, Eccles Street, Dublin 7, Ireland; Gynaecologic Medical Oncology Service, Memorial Sloan-Kettering Cancer Centre, 300 East 66th Street, New York, NY 10065 USA; Department of Histopathology, Mater Misericordiae University Hospital, Eccles Street, Dublin 7, Ireland; Department of Orthopaedic Surgery, Cappagh National Orthopaedic Hospital, Finglas, Dublin 11, Ireland; Department of Radiology, Mater Misericordiae University Hospital, Eccles Street, Dublin 7, Ireland

**Keywords:** Adult osteosarcoma, Neoadjuvant chemotherapy, Poor necrosis rates, MAP, Ifosfamide/etoposide

## Abstract

**Background:**

Chemotherapy in the multimodality treatment of osteosarcoma has improved survival. Reported outcomes on adult patients are limited. Poor necrosis rates post neoadjuvant chemotherapy (NAC) is considered an adverse prognostic factor and attempts have been made to improve survival in this group.

**Patients and methods:**

Adult and young adult patients diagnosed with osteosarcoma between January 1986 and August 2012 were retrospectively reviewed. Patients identified were stratified according to stage (localised or metastatic) and age (≤40 and >40 years). Event free survival (EFS) and overall survival (OS) outcomes were determined. In patients with localised disease ≤40 years, survival was assessed according to necrosis rates post NAC (<90 and ≥90%). NAC consisted of two cycles of methotrexate alternating with doxorubicin/cisplatin (MAP) followed by definitive surgery. Those with ≥90% tumour necrosis continued on MAP. Patients with <90% necrosis received ifosfamide and etoposide (IE) post operatively.

**Results:**

A total of 108 patients were reviewed and 97 were included. Median age was 23 years (range 16–75) and 70% of patients were male. Five year EFS and OS across all groups was 57% and 63% respectively. Of the patients with localised disease (*N* = 81), 5-year overall survival (OS), with a median follow up of 7 years (2–26) was 70% (*p* < 0.0001). Patients aged 16–40 (*N* = 68) with localised osteosarcoma had a significantly improved 5-year OS (74%) compared to those >40 years *(N* = 13) (42%) (*p* = 0.004). Of the 68 patients with localised osteosarcoma ≤40 years, 62 were evaluated according to necrosis rates post MAP. In 33 patients who achieved ≥90% necrosis and continued MAP, 5-year OS was 82%. In 29 patients who had <90% tumour necrosis and received adjuvant IE, 5-year OS was 68% (*p* = 0.15). Multivariate analysis confirmed age and stage as prognostic factors but not poor necrosis rates in our treated population.

**Conclusions:**

Long-term survival outcomes in a predominantly adult Irish population are similar to large reported trials. Age and stage at diagnosis are prognostic. Postoperative ifosfamide/etoposide alone in patients with poor necrosis rates is a feasible regimen, but its role in the adjuvant setting remains uncertain.

**Electronic supplementary material:**

The online version of this article (doi:10.1186/s13569-015-0032-0) contains supplementary material, which is available to authorized users.

## Background

Osteosarcoma is a rare tumour type occurring with an incidence of 0.2–3/1,00,000 per year in Europe [1]. Despite this, it is the most common primary tumour of bone (excluding multiple myeloma), which occurs with a bimodal age distribution. The first peak, in adolescents and young adults, possibly coincides with the pubertal growth spurt. The second peak in patients over 65 years may result from the development of osteosarcoma as a secondary malignancy [2, 3]. The metaphyses of long bones are the most commonly affected site [3] with more than 50% of occurrences adjacent to the knee joint [4].

Prior to the 1970s surgery alone cured <20% of patients; the majority of deaths resulted from the rapid development of lung metastases [5]. Since the incorporation of chemotherapy, multimodality treatment has improved survival to approximately 70% at 5 years with little improvement in the last two decades [4, 6, 7]. Trials demonstrating this survival have largely included paediatric patients and outcomes in adult patients alone are limited.

Where feasible, the standard of care for patients with localised extremity osteosarcoma involves NAC followed by definitive surgical resection and adjuvant chemotherapy [8]. Although NAC versus immediate surgery and adjuvant chemotherapy has not been shown to improve OS [6], it does allow for optimal surgical planning [9] and avoids the need for amputation in most patients. Chemotherapy protocols have generally incorporated four active agents: Methotrexate with leucovorin rescue, doxorubicin, cisplatin and ifosfamide [10]. The most appropriate combination of these drugs however, continues to be debated [8].

NAC also provides prognostic information on tumour necrosis rates, which has been used to tailor postoperative treatment. Based on the Huvos grading system, patients with ≥90% necrosis are considered good responders and those with <90% necrosis as poor responders [11]. Large trials have demonstrated inferior 5-year survival rates in patients with a poor response versus those with a good response (45–56% versus 71–80% respectively) [4, 12–14]. Attempts in the past have failed to improve survival by modifying adjuvant treatment in poor responders and a recent meta-analysis found no benefit in switching or intensifying drugs [7, 15, 16]. Ifosfamide/etoposide (IE) has been shown to have efficacy in the metastatic setting [17] and is considered safe when added to MAP [18]. MAPIE formed the adjuvant arm of poor responders in the EURAMOS-1 trial and provisional results reveal no improvement in survival [19]. At our institution patients with poor necrosis rates have been receiving postoperative IE alone since 1986.

In this article we review the outcomes of a predominantly adult population over a 26-year period and assess the impact of switching chemotherapy to IE in patients with <90% necrosis rates post neoadjuvant MAP.

## Patients and methods

### Patient population

A retrospective review was undertaken of patients with a diagnosis of osteosarcoma seen by the oncology department at the Mater Misericordiae University Hospital from January 1986 to August 2012. This hospital has been a tertiary referral centre for the systemic treatment of adult osteosarcoma, collaborating with Cappagh Hospital, the national orthopaedic hospital, where the majority of bone tumour surgeries are performed. All pathology was reviewed at our institution. During the period analysed, three orthopaedic surgeons performed 98% of the surgeries and the same oncologist delivered systemic treatment in all patients.

Patient details were obtained from the pathology department database and corroborated with the hospital information system search for patients with a diagnosis of osteosarcoma. Medical records of the patients collected were then appraised. Data was obtained on all patients with a histological diagnosis of high-grade osteosarcoma. We included patients with extremity or truncal high-grade osteosarcoma who received systemic treatment. Patients with craniofacial osteosarcoma, which is considered less aggressive, and those who did not receive chemotherapy were excluded. Patients were stratified according to stage: localised and metastatic disease at presentation and then further classified by age: ≤40 and >40 years. Those patients with localised disease ≤40 years, who received NAC were subsequently assessed according to necrosis rates based on the Huvos grading system [11]: poor responders(<90% necrosis, grade I–II) and good responders (≥90% necrosis, grade III–IV).

### Staging and treatment details

Staging was performed using a plain film, radionuclide bone scan and/or magnetic resonance imaging (MRI) of the affected limb. Computed tomography (CT) thorax, abdomen and pelvis (TAP) and Chest X-Ray (CXR) completed radiological staging. More recently FDG PET/CT has been incorporated into routine work up. Full blood count and biochemical analyses were also measured at diagnosis and throughout treatment. The intended operating surgeon performed diagnostic biopsies where possible. All patients were discussed at our institutional multidisciplinary team meeting (MDT) at diagnosis, post NAC and post resection.

The systemic treatment of resectable osteosarcoma at our institution consists of two cycles of high dose methotrexate 8 g/m^2^ alternating with doxorubicin 25 mg/m^2^ days 1–3 and cisplatin 100 mg/m^2^ on day 1 (MAP), followed by definitive surgery and adjuvant chemotherapy (Additional file 1: Table S1). Patients with tumours which upon histological review demonstrate ≥90% necrosis continue MAP for a further two alternating cycles while those who have <90% necrosis rates switch to ifosfamide 1,800 mg/m^2^ with mesna 1,800 mg/m^2^ days 1–5 and etoposide 100 mg/m^2^ days 1–5 for a total of four cycles (Additional file 1: Table S1). Dosing and scheduling of chemotherapy administered was confirmed with the pharmacy department.

Patients with metastases at diagnosis who were considered resectable were treated with the same protocol and at surgery had resection of the primary lesion and metastases where applicable.

Close surveillance was initiated upon completion of adjuvant treatment. Patients were seen 3 monthly for the first 2 years with a CXR; a CT Thorax was performed every 6 months. Thereafter patients were reviewed 4–6 monthly with a CXR until year 5 at which point annual follow-up was commenced.

### Data collection

Demographic and clinical details were extracted from the patient’s medical records. Information on histology was obtained from the laboratory report system. Data collected included age, sex, stage at diagnosis, tumour site, type of operation, percentage necrosis, chemotherapy regimen used and number of cycles administered. Relapse date and site, together with survival and follow up information was also recorded.

### Statistical analysis

Demographic details and treatment variables are reported by descriptive statistics. EFS and OS were computed from time of tissue biopsy until first recurrence or death respectively. Time of cut off for analyses was July 31st 2014. The Kaplan–Meier survival analysis method and the log rank test were used to estimate survival probability and compare survival within stratified groups. The Cox Regression model was used to assess the prognostic impact of age, stage and necrosis rates on survival. Analyses were conducted using SPSS 20.0

## Results

### Patient characteristics

Between January 1st 1986 and July 31st 2012 108 adult patients with a diagnosis of osteosarcoma were reviewed by medical oncology. Eight patients who did not receive chemotherapy due to poor performance status (median age 73 years, range 46–81) and three patients with craniofacial disease were excluded. A total of 97 patients were included. Detailed baseline patient demographics and tumour characteristics are available in Additional file 2: Table S2. Specific high-grade subtypes were incompletely reported during the time period and not available for analysis. Five patients were under 18 years (16–17 years) with the majority (95%) ≥18 years. Median age overall was 23 years (16–73). Seventy percent of patients were male. The most common site of tumour was the femur (54%) followed by the tibia (20%). Of the total patients included (*N* = 97) 81 (84%) had localised disease and 16 (16%) had metastatic osteosarcoma at diagnosis. In patients with localised disease, 68/81 (84%) were ≤40 years (16–40) and in patients with metastatic disease at diagnosis, 11/16 (68%) were ≤40 years (17–40). Follow-up for all patients ranged from 2 to 28.6 years with a median of 7 years.

### Surgical treatment

Limb-salvage surgery was performed in 70% of patients with disease of the extremities (*N* = 90). Of these 90 patients 77 had localised disease and 13 metastatic disease. Limb-salvage was possible in 77% of patients with localised disease and was performed in only 31% of patients with metastatic disease (4/13) (Additional file 2: Table S2). Notably the period of this review from 1986 to 2012 has seen changes in approaches to the surgical management of extremity osteosarcoma with the increasing use of limb-salvage techniques in recent years [20, 21].

### Chemotherapy

In patients with localised disease (*N* = 81), 618 of planned 648 chemotherapy cycles were completed (95%). Eight patients (10%) did not complete the intended cycles of treatment. Four patients ≥40 years with localised disease, developed grade 3–4 toxicities, as did three patients ≤40 years. One further patient ≤40 years refused two final cycles of adjuvant treatment. Of the 16 patients with metastatic disease 11 (70%) completed intended first line treatment.

### Survival outcomes

**Figure 1:**
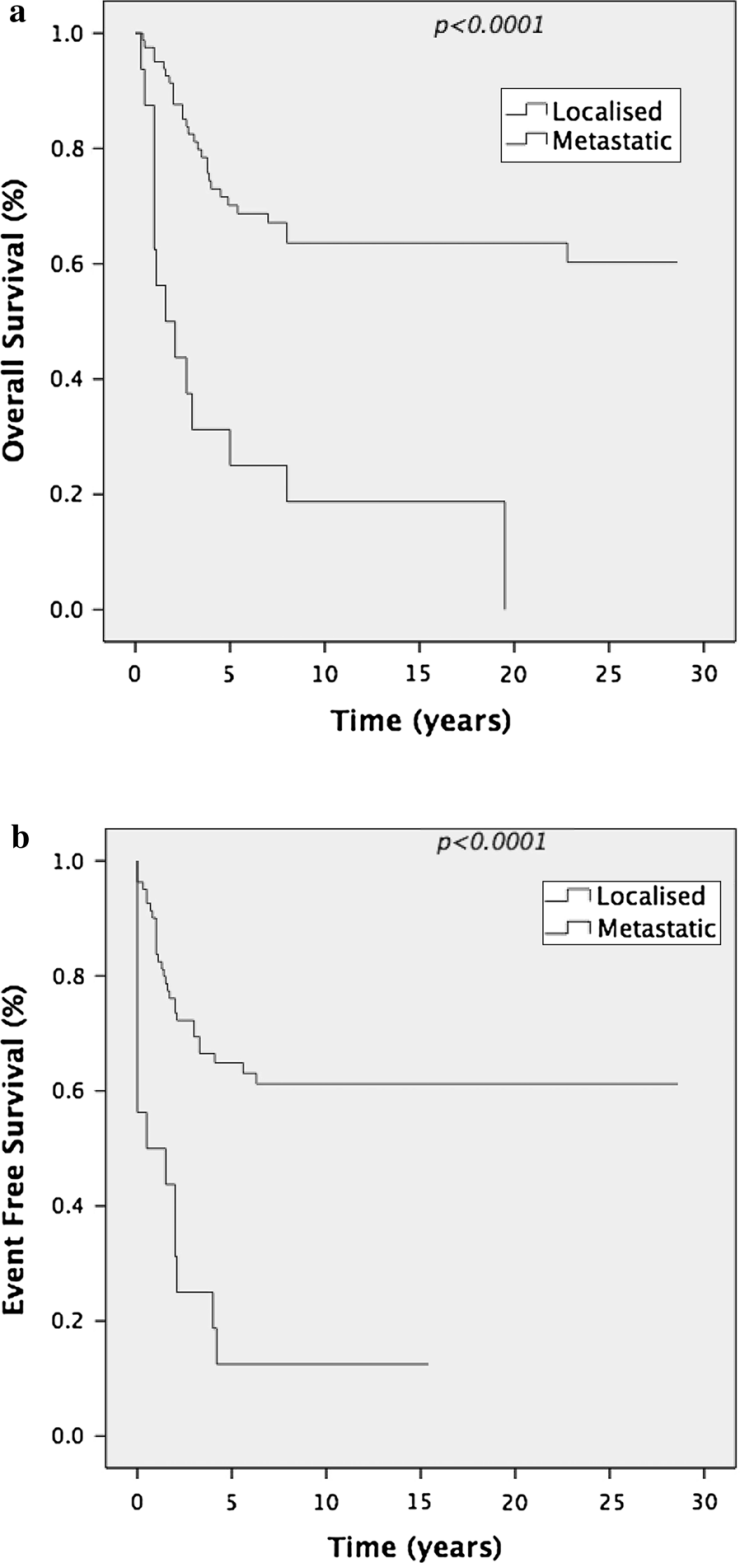
**a** Overall survival in patients with localised disease versus metastatic disease. **b** Event free survival in patients with localised disease versus metastatic disease.

**Figure 2:**
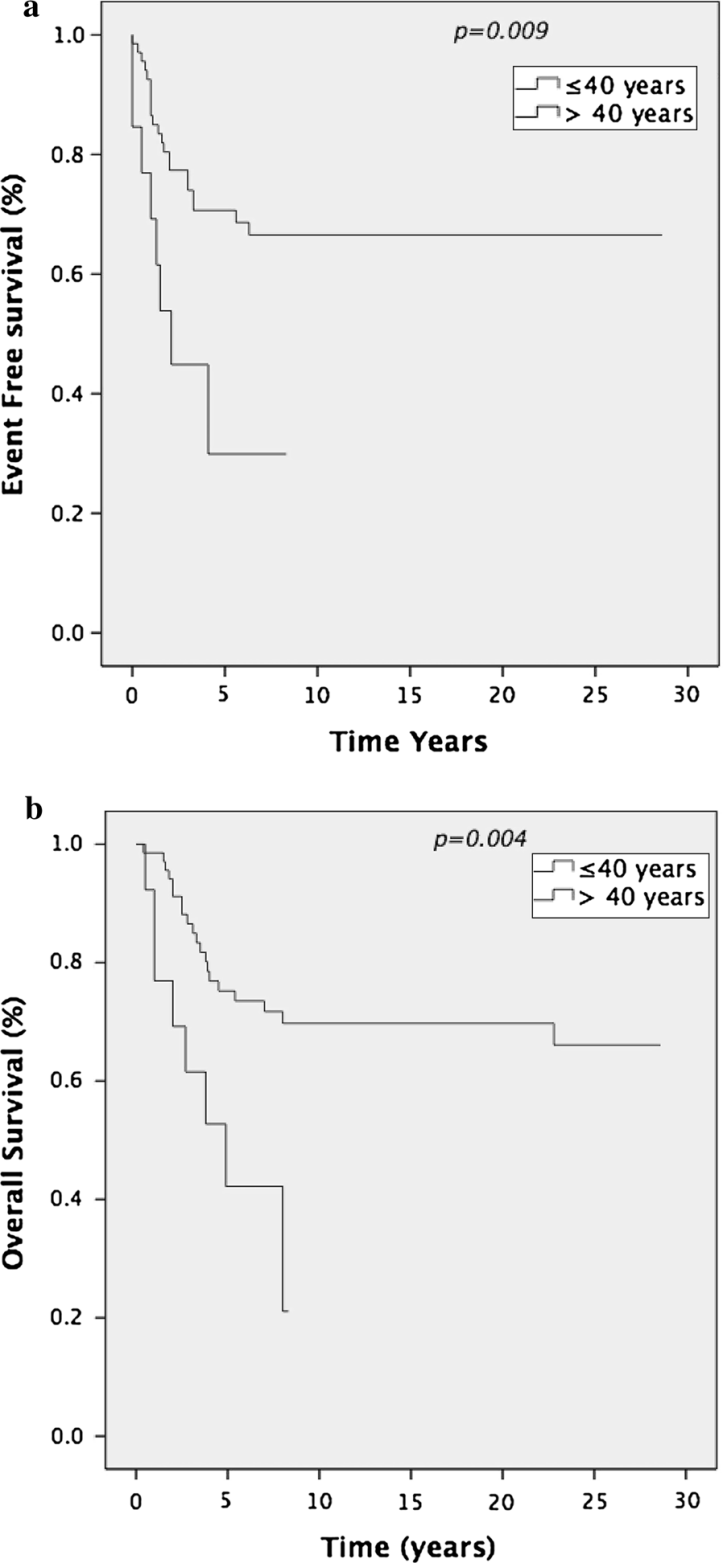
**a** EFS and **b** OS in localised disease according to age.

The 5-year EFS and OS across all groups was 57 and 63% respectively. Stage at diagnosis was prognostic. Patients with localised disease (*N* = 81) had a significantly improved 5-year OS compared to those with metastatic disease at diagnosis (*N* = 16), 70 versus 25% *p* < 0.0001 (all age groups) (Figure 1a). EFS was 65 versus 13% *p* < 0.0001 (Figure 1b). Despite unbalanced numbers in each group age was also prognostic. EFS in localised disease ≤40 years (*N* = 68) was 70% compared to 30% in those >40 years (*N* = 13) (*p* = 0.009). Corresponding 5-year OS was 74% compared to 42%. (*p* = 0.004) (Figure 2a, b). Seven of the 13 patients >40 years (median age 54 years, range 44–74) with localised disease have died. Of these four were unable to complete chemotherapy due to toxicity. Multivariate analysis using the Cox Regression model confirmed the prognostic impact of age and stage for EFS (*p* = 0.001 and *p* = 0.001 respectively) and for OS (*p* = 0.002 and *p* = 0.001 respectively).

### Relapsed disease in patients with localised osteosarcoma at diagnosis

Of the 81 patients with localised disease, 32 (40%) experienced recurrences. This included seven patients >40 years (54% of all patients >40 years). The lung was site of first recurrence in 22/32 (69%) patients, with locally recurrent disease in nine patients. Three of these nine patients developed lung metastases within 1 year and one patient developed lung metastases 4 years later. One patient developed brain metastases as first documented recurrence but was also found to have lung metastases post complete imaging. Of the 32 patients who recurred, 11 patients (9 ≤ 40 years and 2 > 40 years) are alive and disease free with a median follow-up of 10.3 years (range 4.8–27.1). Four of these 11 patients developed local recurrences and were treated with surgery including one amputation; the remaining seven patients had thoracotomies for lung metastases (median number of thoracotomies 1; range 1–3).

### Impact of switching chemotherapy in patients with poor necrosis (localised ≤40 years)

Of the 68 patients with localised disease ≤40 years, 62 were eligible for assessment of survival according to necrosis rates. The remaining six patients had upfront surgery without NAC and were thus excluded. Five of these six patients were initially thought to have parosteal osteosarcoma and one patient a chondrosarcoma. Pathological review of tumour specimens post resection revealed high-grade osteosarcoma. These six patients all received eight cycles of chemotherapy in the adjuvant setting and five are still alive with a median follow up of 3.8 years (2.2–14.4).

**Figure 3:**
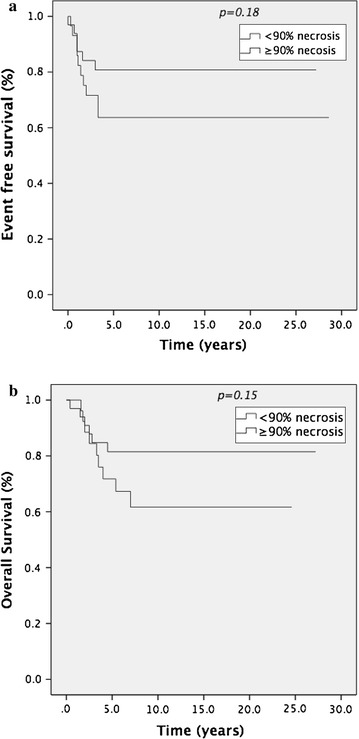
**a** EFS and **b** OS in patients with localised disease <40 years according to necrosis rates.

The 62 patients included all had initial biopsies and post NAC histology reviewed at our institution. Median follow-up time in this group was 9.4 years (range 2–28.6). 33 patients demonstrated ≥90% necrosis post MAP and 29 patients <90% necrosis. There was no significant difference in 5 year-EFS in good responders (81%) and in poor responders (64%) (*p* = 0.18) (Figure 3a). Five year OS was 82% in those with ≥90% necrosis versus 68% in those with <90% necrosis; this result was again not statistically significant *p* = 0.15 (Figure 3b). When included in multivariate analysis, necrosis rates post NAC was not prognostic for EFS (*p* = 0.9) or OS (*p* = 0.62) in our cohort (Additional file 3: Table S3).

### Outcomes in patients with metastatic disease at diagnosis

Sixteen patients (16%) had evidence of metastases at diagnosis and 5-year OS was 25%. Lung metastases were present in 13 patients and 3 patients had multifocal disease. Of the 13 patients with lung metastases 7 had thoracotomies, 2 at primary resection and 5 at recurrence. The median number of thoracotomies was 1 (range 1–4). Two patients with metastases at presentation, 1 with multifocal disease and 1 with lung metastases are still alive 8.3 and 15.4 years later.

## Discussion

This is a retrospective study of predominantly adult osteosarcoma patients treated systemically at a single institution over 26 years. We have established that long term overall survival outcomes in this Irish population are comparable to previously reported large clinical trials. This study has also demonstrated the feasibility of IE postoperatively in patients with a poor response to neoadjuvant MAP.

Adult patients are generally underrepresented in osteosarcoma trials despite approximately half of patient diagnoses occurring in those over 20 years. [22] The median age in our study was 23 years with 95% of patients ≥18 years and 70% of patients included were male. The male preponderance in osteosarcoma is well documented and although the proportion is slightly higher than reported ratios [3, 23] this may be explained by the older age of the patients included, as peak incidence in the female population occurs earlier [23].

Inferior outcomes have been reported in patients over 40 [24, 25] and over 65 years [26]. A meta-analysis reported by Harting et al. investigating age as a prognostic factor in osteosarcoma, revealed that patients aged 21–40 (*N* = 110) had the same outcomes as that of the paediatric population with inferior survival in those >40 years (*N* = 56) [27]. This study acknowledges however that age alone is unlikely to contribute to inferior outcomes, but rather site, size and tumour necrosis rates in this group. In our study 5-year OS for all 97 patients included was 64%, improving to 74% when selecting patients aged 16–40 years with localised disease (*N* = 68). A small number of patients (*N* = 18) were >40 years at diagnosis. Of these, patients with localised disease at presentation (*N* = 13) had a significantly inferior survival at 5 years (45%) compared to patients ≤40 years. Seven of thirteen patients died and in four of these less than 50% of chemotherapy cycles were completed as a result of toxicity. The remaining three patients all had tumours >8 cm, one of whom had positive surgical margins; all three demonstrated poor necrosis rates post NAC. These factors are likely to have impacted on survival, however numbers are too small to make further conclusions. One of the largest studies in patients over 40 years reported by Grimer et al. [25] describes 238 patients with non metastatic osteosarcoma and reports a 5-year survival of 46%. Other studies report superior survival rates of 55–62% at 5 years [4, 28]. It is therefore likely that other patient and tumour characteristics contribute to outcomes.

Less than 20% of patients with osteosarcoma will present with de novo metastatic disease [29]. In our cohort 16/97 (16%) patients had metastatic disease at diagnosis. The 5-year OS of 25% in this group is consistent with previous studies, which highlight number and resectability of metastases as prognostic in those with de novo metastatic disease [29, 30]. Of patients with localised disease at diagnosis who recurred in our study, 11/32 (34%) were salvaged with surgery. Seven of these patients were rendered disease free post thoracotomies. The aggressive surgical management of recurrent osteosarcoma with complete clearance is necessary for long-term survival and is well described [31–33]. Duration of relapse free interval and number of lesions are considered prognostic factors [31].

When evaluating the impact of poor necrosis rates on survival we were able to include 62 patients with localised disease ≤40 years who received NAC. Responsiveness to NAC has been established as a predictor of survival [4, 12–14, 34].

The COSS, EOI and IOR groups have reported 5-year survival rates of 55.5, 45 and 56% respectively for poor responders with localised osteosarcoma [4, 12, 35]. Multiple studies have attempted to improve outcomes in this poor prognostic group by switching drugs or intensifying treatment, however a meta-analysis published by Anninga et al. [16] showed no benefit to either method.

MAP as a neoadjuvant regimen has been used since the 1980s and is considered standard of care in the United States [36]. This combination formed the backbone of the international multi-group EURAMOS-1 trial [37], the largest trial conducted in osteosarcoma. Survival outcomes in osteosarcoma have plateaued in recent decades and this group attempted to improve outcomes in both good and poor responders. The addition of IFN alpha-2b to MAP in good responders has recently been reported and failed to enhance survival [38]. Patients with poor necrosis rates were randomized to remain on MAP postoperatively or switch to MAP and IE.

The role of ifosfamide in osteosarcoma has been controversial. It has been shown to be active in the metastatic setting [17, 39] and has shown improvements in recurrence free survival and overall survival in patients at first recurrence [32]. The use of ifosfamide as a fourth drug in the neoadjuvant setting however does not improve histologic response and increases hematological toxicity [40]. When IE is added to MAP in poor responders post operatively results have differed. The IOR group have found favorable outcomes with use of ifosfamide [41] however the Scandinavian Sarcoma Group (SSG) failed to show a benefit of the addition of IE to MAP adjuvantly in poor responders [42]. Early results from EURAMOS-1 have also failed to show a benefit of additional IE to MAP in poor necrosis patients. It should be noted that fewer patients in this group received the intended doses and more toxicities were experienced [19]. In our patients survival at 5 years was 82% in good responders versus 68% in those treated with IE alone postoperatively. This difference was not statistically significant but suggests that IE is an acceptable adjuvant regimen in patients with poor necrosis rates.

## Conclusions

Adult and young adult osteosarcoma patients under 40 years with localised osteosarcoma treated at our institution have equivalent outcomes compared to paediatric patients. Patients over 40 years are more challenging and are more likely to experience toxicity. Switching chemotherapy to ifosfamide/etoposide alone in patients with poor necrosis rates post neoadjuvant methotrexate, doxorubicin, cisplatin is a feasible regimen, but where these drugs fit in the treatment paradigm remains to be established.

## Additional files

Additional file 1:
**Table S1.** Chemotherapy protocol for resectable disease. Additional file 2:
**Table S2.** Patient characteristics. Additional file 3:
**Table S3.** Multivariate Cox-regression analysis of prognostic factors for event free survival and overall survival.
