# Supplementary material for: Perioperative chemotherapy in the treatment of osteosarcoma: a 26-year single institution review
Source: Clin Sarcoma Res. 2015 Jul 14;5:17. doi: 10.1186/s13569-015-0032-0 (PMC4501053; doi:10.1186/s13569-015-0032-0)
Supplement: Additional file 1: — Table S1. Chemotherapy protocol for resectable disease. [file 13569_2015_32_MOESM1_ESM.docx]

| Day of administration | 1 | 11-13 | 33 | 43-45 | 64 | 78 | 89-91 | 110 | 120-122 |
| --- | --- | --- | --- | --- | --- | --- | --- | --- | --- |
|  | | | | | S  U  R  G  E  R  Y | **If** ≥ **90% necrosis** | | | |
| *Methotrexate 8g/m2* | **x** |  | **x** |  |  | **x** |  | **x** |  |
| *Calcium leucovorin 30mg/m2* | **x** |  | **x** |  |  | **x** |  | **x** |  |
| *Doxorubicin 25mg/m2* |  | **xxx** |  | **xxx** |  |  | **xxx** |  | **xxx** |
| *Cisplatin 100mg/m2* |  | **x** |  | **x** |  |  | **x** |  | **x** |
|  | | | | |  | **If <90% necrosis** | | | |
| Day of administration |  |  |  |  |  | **78-82** | **99-103** | **120-124** | **141-145** |
| *Ifosfamide 1800mg/m2* |  |  |  |  |  | **xxxxx** | **xxxxx** | **xxxxx** | **xxxxx** |
| *Mesna 1800mg/m2* |  |  |  |  |  | **xxxxx** | **xxxxx** | **xxxxx** | **xxxxx** |
| *Etoposide 100mg/m2* |  |  |  |  |  | **xxxxx** | **xxxxx** | **xxxxx** | **xxxxx** |

**Table S1. *Chemotherapy protocol for resectable disease***
