# Supplementary material for: Perioperative chemotherapy in the treatment of osteosarcoma: a 26-year single institution review
Source: Clin Sarcoma Res. 2015 Jul 14;5:17. doi: 10.1186/s13569-015-0032-0 (PMC4501053; doi:10.1186/s13569-015-0032-0)
Supplement: Additional file 2: — Table S2. Patient characteristics. [file 13569_2015_32_MOESM2_ESM.docx]

|  |  | *N* =97 (%total) | Localised (*N*=81) | | Metastatic  (*N*=16) | |
| --- | --- | --- | --- | --- | --- | --- |
| Sex | Male | 68 (70%) | 56 | | 12 | |
|  | Female | 29 (30%) | 25 | | 4 | |
| Age | ≤40years | 79 (84%) | 68 | | 11 | |
|  | >40years | 18 (16%) | 13 | | 5 | |
| Site | Extremity | 90 (93%) | ≤40yrs | >40yrs | ≤40yrs | >40yrs |
|  | *Femur* | 52 (54%) | 38 | 6 | 5 | 2 |
|  | *Tibia* | 19 (20%) | 18 | 1 | 0 | 0 |
|  | *Humerus* | 13 (13%) | 6 | 4 | 3 | 0 |
|  | *Ulna* | 3 (3%) | 2 | 1 | 0 | 0 |
|  | *Fibula* | 2 (2%) | 0 | 0 | 2 | 0 |
|  | *Calcaneus* | 1 (1%) | 0 | 1 | 0 | 0 |
|  |  |  |  |  |  |  |
|  | Non Extremity | 7 (7%) |  |  |  |  |
|  | *Pelvic* | 2 (2%) | 2 | 0 | 0 | 0 |
|  | *Spinal* | 3 (3%) | 1 | 0 | 0 | 2 |
|  | *Scapula* | 2 (2%) | 1 | 0 | 0 | 1 |
| Surgery (extremity N=90) | Limb salvage | 63 (70%) | 51 | 8 | 3 | 1 |
|  | Amputation | 27 (30%) | 14 | 4 | 8 | 1 |

***Table S2: Patient characteristics***
