# Supplementary material for: Perioperative chemotherapy in the treatment of osteosarcoma: a 26-year single institution review
Source: Clin Sarcoma Res. 2015 Jul 14;5:17. doi: 10.1186/s13569-015-0032-0 (PMC4501053; doi:10.1186/s13569-015-0032-0)
Supplement: Additional file 3: — Table S3. Multivariate Cox-regression analysis of prognostic factors for event free survival and overall survival. [file 13569_2015_32_MOESM3_ESM.docx]

| Variable | OS  Hazard Ratio (95% CI) | P vaue | EFS  Hazard Ratio  (95% CI) | P Value |
| --- | --- | --- | --- | --- |
| Stage  (localised vs metastatic) | 3.9  (1.6-9.3) | 0.002 | 3.7  (1.7-8.2) | 0.001 |
| Age  ≤40yrs;>40yrs | 4.2  (1.9-9.3) | 0.001 | 4.0  (1.7-9.4) | 0.001 |
| Necrosis rates  <90%, ≥90% | 0.83  (0.40-1.74) | 0.62 | 0.97  (0.47-2.00) | 0.92 |

Table S3: Multivariate Cox-regression analysis of prognostic factors for event free survival and overall survival.
